# Arhgap21 Deficiency Results in Increase of Osteoblastic Lineage Cells in the Murine Bone Marrow Microenvironment

**DOI:** 10.3389/fcell.2021.718560

**Published:** 2021-11-30

**Authors:** Mariana Ferreira Pissarra, Cristiane Okuda Torello, Rafael Gonçalves Barbosa Gomes, Rodrigo Naoto Shiraishi, Irene Santos, Karla Priscila Vieira Ferro, Matheus Rodrigues Lopes, Patricia Maria Bergamo Favaro, Sara Teresinha Olalla Saad, Mariana Lazarini

**Affiliations:** ^1^ Hematology and Hemotherapy Center, University of Campinas, São Paulo, Brazil; ^2^ Institute of Environmental, Chemical and Pharmaceutical Sciences—Federal University of São Paulo, São Paulo, Brazil

**Keywords:** osteocalcin, myelodysplastic syndromes, acute myeloid leukemia, RhoGAP, Rho GTPase, RhoA, Cdc42 (cell division cycle 42 GTP-binding protein)

## Abstract

ARHGAP21 is a member of the RhoGAP family of proteins involved in cell growth, differentiation, and adhesion. We have previously shown that the heterozygous Arhgap21 knockout mouse model (Arhgap21^+/−^) presents several alterations in the hematopoietic compartment, including increased frequency of hematopoietic stem and progenitor cells (HSPC) with impaired adhesion *in vitro*, increased mobilization to peripheral blood, and decreased engraftment after bone marrow transplantation. Although these HSPC functions strongly depend on their interactions with the components of the bone marrow (BM) niche, the role of ARHGAP21 in the marrow microenvironment has not yet been explored. In this study, we investigated the composition and function of the BM microenvironment in Arhgap21^+/−^ mice. The BM of Arhgap21^+/−^ mice presented a significant increase in the frequency of phenotypic osteoblastic lineage cells, with no differences in the frequencies of multipotent stromal cells or endothelial cells when compared to the BM of wild type mice. Arhgap21^+/−^ BM cells had increased capacity of generating osteogenic colony-forming units (CFU-OB) *in vitro* and higher levels of osteocalcin were detected in the Arhgap21^+/−^ BM supernatant. Increased expression of *Col1a1*, *Ocn* and decreased expression of *Trap1* were observed after osteogenic differentiation of Arhgap21^+/−^ BM cells. In addition, Arhgap21^+/−^ mice recipients of normal BM cells showed decreased leucocyte numbers during transplantation recovery. Our data suggest participation of ARHGAP21 in the balanced composition of the BM microenvironment through the regulation of osteogenic differentiation.

## Introduction

Normal hematopoiesis is controlled by the dynamic interactions between hematopoietic stem cells (HSC) and specialized microenvironments (niches) composed of different cell types within the bone marrow (BM) (Galán-Díez and Kousteni 2017; Méndez-Ferrer et al., 2020). Endothelial cells, mesenchymal cells (MSCs), and osteoblasts are key components of BM niches, acting as regulators of hematopoiesis (Méndez-Ferrer et al., 2010, 2020; Pinho and Frenette 2019). Disruptions in BM niches have been found in hematological malignancies (Krevvata et al., 2014; Wang et al., 2016; Galán-Díez and Kousteni 2017; Méndez-Ferrer et al., 2020) and there is increasing evidence that hematopoietic malignant cells actively remodel their local microenvironment to subserve their physiological demands. For instance, in myeloproliferative neoplasia, BM-MSCs were stimulated by malignant cells to produce altered osteoblasts, transforming the BM microenvironment into a leukemic niche (Schepers et al., 2013). Conversely, changes in specific components of the BM niche may result in hematological malignancies (Man et al., 2021). Osteoblasts have been directly implicated in the development of myeloid disorders in mice models of myelodysplasia and myeloproliferative neoplasms leading to acute myeloid leukemia (AML) (Krevvata et al., 2014). Thus, identifying new molecules involved in the regulation of the BM niche presents an unexploited opportunity for therapeutic targeting.

Members of the Rho family of GTPases participate in the regulation of normal and malignant hematopoiesis, including interactions between HSC and the BM microenvironment (Yang et al., 2007; Cancelas and Williams 2009). Rho GTPases comprise small G-proteins well known for their functions in controlling cytoskeletal rearrangements. Most Rho GTPases cycle between active and inactive conformations (GTP/GDP-bound) aided by three types of proteins: guanine exchange factors, GDP dissociation inhibitors and GTPase-activating proteins (GAPs) (McMichael et al., 2017). RhoGAPs stimulate the conversion from the GTP-bound form to the GDP-bound form, acting as negative regulators of Rho GTPases.

Rho GTPase deregulation found in abnormal hematopoiesis frequently results from changes in RhoGAPs. Heterozygous knockout mouse model for p190 RhoGAP exhibit altered constitution of the BM microenvironment, with an increased number of adipocytes and decreased number and functionality of osteoblasts (Raman et al., 2013). Knockdown of the RhoGAP ARHGAP18 in murine MSCs increases RhoA activity with concomitant suppression of adipogenesis and increase in osteogenic commitment (Thompson et al., 2018). The Arhgap28 knockout mice showed normal bone phenotype, but with decreased expression of genes that encode extracellular matrix proteins (Yeung et al., 2014).

Our group has been studying the role of the RhoGAP protein ARHGAP21 in hematopoietic and non-hematopoietic cells (Sanchez Bassères et al., 2002; Barcellos et al., 2013; Lazarini et al., 2013; Xavier-Ferrucio et al., 2018; Bernusso et al., 2021). ARHGAP21 presents RhoGAP activity for the Rho GTPases RhoA, RhoC and Cdc42 (Dubois et al., 2005; Lazarini et al., 2013) and interacts with FAK, α-tubulin and β-arrestin (Bigarella et al., 2009; Anthony et al., 2011; Bernusso et al., 2021). Upregulation of ARHGAP21 was detected during erythrocytic, granulocytic (Sanchez Bassères et al., 2002) and megakaryocytic differentiation (Bernusso et al., 2021), and its silencing in human hematopoietic progenitor cells decreased erythroid commitment (Xavier-Ferrucio et al., 2018). Heterozygous mouse model with reduced Arhgap21 expression (Arhgap21^+/−^) showed increased frequency of hematopoietic stem and progenitor cells, but with impaired functionality. Arhgap21^+/−^ hematopoietic progenitor cells also presented defective adhesion and enhanced mobilization (Xavier-Ferrucio et al., 2018). Accelerated hemostatic response was another feature of Arhgap21^+/−^ mice (Bernusso et al., 2021). Despite all these hematological changes, the impact of ARHGAP21 reduction in the BM microenvironment had not been investigated. In this study, we characterized the cellular composition of Arhgap21^+/−^ mice BM microenvironment and the impact of Arhgap21 reduction on osteoblast differentiation of BM MSCs.

## Materials and Methods

### Heterozygous Arhgap21 Mouse (Arhgap21^+/−^)

Arhgap21 heterozygous mice (Arhgap21^+/−^) were obtained by Arhgap21^+/−^ versus wild type (WT) crossed mating pairs, according to Mendelian distribution. All animals were bred and maintained at the University of Campinas (UNICAMP) and housed four per cage. Environmental conditions were temperature-controlled (21 ± 2°C), 55 ± 5% humidity and a 12 h/12 h light-dark circadian cycle with access to food and water *ad libitum*. Arhgap21^+/−^ mice present an average of approximately 50% reduction of Arhgap21 mRNA and protein levels compared to WT, and their lifespan is similar to that of WT mice (Xavier-Ferrucio et al., 2018). Arhgap21^+/−^ mice and WT littermates (control) at 8–12-weeks of age were used in all experiments. All procedures were approved by the Institutional Animal Experimentation Ethics Committee (CEUA 4894-1/2018/UNICAMP) and conducted according to National Institutes of Health Guide for the Care and Use of Laboratory Animals.

### Obtention of BM Cells and Supernatant

BM cells were collected from the femur and tibiae of Arhgap21^+/−^ and WT mice by flushing. The flushing method consists of cutting the ends of the bones and removing the bone marrow cells using a syringe with 25-gauge needle and 200 µl of phosphate-buffered saline (PBS) (Huang et al., 2015). Cells were centrifuged at 1,500 rpm for 5 min and BM supernatant was then collected and stocked at −20°C for protein analysis.

#### Immunophenotyping of Endothelial, Multipotent Stromal and Osteoblastic Lineage Cells

A total of 1 × 10^7^ BM cells collected as described above were resuspended in 100 µl of PBS containing specific anti-mouse antibodies (Lin/APC-BD Biosciences, CD45/PerCP-Cy5.5-Biolegend, CD31/FITC-Biolegend, Sca-1/PE-Cy7-Biolegend and CD51/PE-Biolegend) and incubated for 30 min at room temperature in the dark. Frequency of each cell subset was determined using a FacsAria II (BD Biosciences) with the following antibodies: Multipotent Stromal Cells—MSCs (CD45-/Lin-/CD31-/Sca1+/CD51+); Osteoblastic Lineage Cells—OBCs (CD45-/Lin-/CD31-/Sca1-/CD51+); Arteriolar Endothelial Cells—AEC (CD45-/Lin-/CD31+/Sca1+) and Sinusoidal Endothelial Cells—SEC (CD45-/Lin-/CD31+/Sca1-). Analysis was performed with FlowJo software (TreeStar Inc.).

### CFU-F and CFU-OB Assay

Colony Forming Unit-Fibroblast (CFU-F) and Colony Forming Unit-Osteoblast (CFU-OB) assays were performed according to Balderman et al., 2016, using three mice per group. Briefly, for CFU-F assays, 10^6^ total BM cells/well were cultured in a six-well dish in Minimum Essential Medium Eagle, alpha modification (alpha MEM) media containing 20% fetal bovine serum (FBS) for 7 days, when fresh media was added. After 14 days, adherent cells were fixed in formalin 10% buffer and stained for crystal violet (0.5% in methanol) or alkaline phosphatase activity (0.005% weight/volume naphthol AS MX-PO4, and 0.03% weight/volume fast red violet LB salt in 100 mM Tris-HCl). For CFU-OB assays, 4 × 10^6^ total BM cells/well were cultured in a six-well dish in alpha MEM media containing 10% FBS for 5–6 days, when non-adherent cells were removed and new mineralizing alpha MEM media (containing 10% FBS, 50 ug/ml L-ascorbic acid 2-phosphate and 10 mM glycerol2-phosphate disodium salt hydrate) was added and replenished every 2–3 days. On the 17th day, cells were fixed with 10% neutral buffered formalin and stained for alkaline phosphatase activity or for von Kossa positive bone nodules (2.5%weight/volume AgNO_3_). Reagents were purchased from Sigma-Aldrich.

### RhoA and Cdc42 Activation Assays

Arhgap21^+/−^ and WT BM cells were induced for osteogenic differentiation as described above and total protein was extracted on days 0, 7 and 16. Three pools of BM cells containing three mice each were evaluated per group. Day 0 denotes the addition of mineralizing media. RhoA and Cdc42 activities were determined in protein extracts using G-Lisa Activation Assays (Cytoskeleton, Inc.).

### Cytokine Quantification

Cytokine levels were determined in peripheral blood (PB) and BM supernatant of Arhgap21^+/−^ and WT mice. Osteocalcin levels were measured using Mouse OC/BGP (Osteocalcin) ELISA Kit, 96T (Elabscience Biotechnology Co.). Levels of G-CSF, M-CSF, IFN-γ, IL-1α, IL-1β, IL-4, IL-10, IL-17, and VEGF-A were measured using the customized Miliplex MAP Mouse Cytokine Magnetic Kit (Milipore, MCYTMAG-70K-PX32). Levels of TGF-β1 were measured by Multi-species TGFb-Singleplex (Milipore, TGFBMAG-64k-01).

### Bone Marrow Transplants

Bone marrow transplantation was performed by injecting normal hematopoietic cells in Arhgap21^+/−^ or WT mice. Briefly, 10^6^ bone marrow cells from B6.SJL/BoyJ (PEP; CD45.1+, Jackson Labs) mice were transplanted into 9.5Gy sub-lethally irradiated WT (*n* = 5) or Arhgap21^+/−^ (*n* = 8) (CD45.2+) recipient mice. All mice were 10-weeks old when transplanted. Donor reconstitution (CD45.1+) and hematological parameters (hemoglobin, platelets, and WBC) were evaluated every 4 weeks after transplant until 16 weeks post-transplantation, when animals were terminated. PB was collected by ocular cavity and hematological parameters were analyzed using a CELL-DYN Emerald Hematology System counter (Abbott Laboratories). Chimerism was evaluated by FACScalibur flow cytometer using mAb CD45.1-PE (BD Bioscience).

### Immunophenotyping of BM and PB Cells From Recipient Mice

BM cells from recipient mice were characterized 16 weeks post-transplant by flow cytometry using the mAbs CD45-PE (pan leucocyte marker); Lineage-APC/Sca-1-PerCP/c-Kit-FITC (LSKs); Gr-1-PerCP/Mac-1-FITC (myeloid cells). PB cells were characterized using CD45-PE (pan leucocyte marker); CD3-FITC (T cells); B220-APC (B cells); CD11b-FITC (monocytes/granulocytes). All antibodies were from BD Biosciences. At least 1,000,000 events were acquired using a FACScalibur flow cytometer. Gated cells were analyzed using the FlowJo software (TreeStar Inc.). Absolute numbers of WBC subpopulations were calculated by multiplying their frequencies per total leucocyte number obtained with complete blood count test.

### Patient Samples

*ARHGAP21* gene expression was analyzed in BM-MSCs cultures from myelodysplastic syndromes (MDS, *n* = 21) and acute myeloid leukemia (AML, *n* = 18) patients. BM-MSCs cultures obtained from heathy donors (HD) were used as control (*n* = 7). Briefly, BM mononuclear cells were isolated by density gradient centrifugation and cultured at 37°C, 5% CO_2_ in Dulbecco’s Modified Eagle Medium (DMEM) media containing 1% penicillin/streptomycin, 1% L-glutamine and 10% FBS for 3–4 days, when non-adherent cells were removed. Monolayers were sub-cultured at approximately 80% confluence. To minimize the possible modifications that these cells may have acquired when cultured, all cell cultures were maintained under the same conditions and expanded only until the fourth passage. MSC cultures were previously characterized by our group (Lopes et al., 2017) and RNA samples were stored at −80°C. ARHGAP21 expression was also evaluated in total BM cells from HD (*n* = 9) and patients with MDS (*n* = 48) and AML (*n* = 50). MDS patients were classified according to the World Health Organization (WHO-2016) and AML patients were classified into AML with myelodysplasia-related changes (AML-MRC) and *de novo* AML. All patients were untreated when samples were collected, and their characteristics are described in Supplementary Table 1. Participants provided their informed written consent, and the study was approved by the Ethics Committee of UNICAMP and was adherent to the Declaration of Helsinki.

### Quantitative PCR

Total RNA was extracted from patient samples using TRIzol Reagent (Thermo Fisher Scientific). RNeasy Micro Kit (Qiagen) was used to isolate total RNA from BM cells under osteoblastic differentiation. RevertAid First Strand cDNA synthesis kit (Thermo Scientific) was used for the reverse transcription reaction of all samples. Gene expression was analyzed by quantitative PCR using Maxima SYBR Green/ROX qPCR Master Mix (2X) (Thermo Scientific) and ABI-7500 Sequence Detection System (Applied Biosystems). A negative “no template control” was included for each pair of primers. Primer sequences are described in Supplementary Table 2. Relative expression was calculated using the 2^−ΔΔCT^ equation (Livak and Schmittgen 2001).

### Statistical Analysis

Statistical analysis was performed using GraphPad Prism 9 software (GraphPad Software). ANOVA or Student *t*-test was used to analyze the results obtained with Arhgap21^+/−^ and WT mice. Mann-Whitney test was used for comparisons of gene expression from patient samples. Data represent mean ± SD. A *p* < 0.05 was considered statistically significant.

## Results

### Arhgap21^+/−^ Mice Present Increased Osteoblastic Lineage Cells (OBCs) and Osteocalcin Levels in the Bone Marrow

**FIGURE 1:**
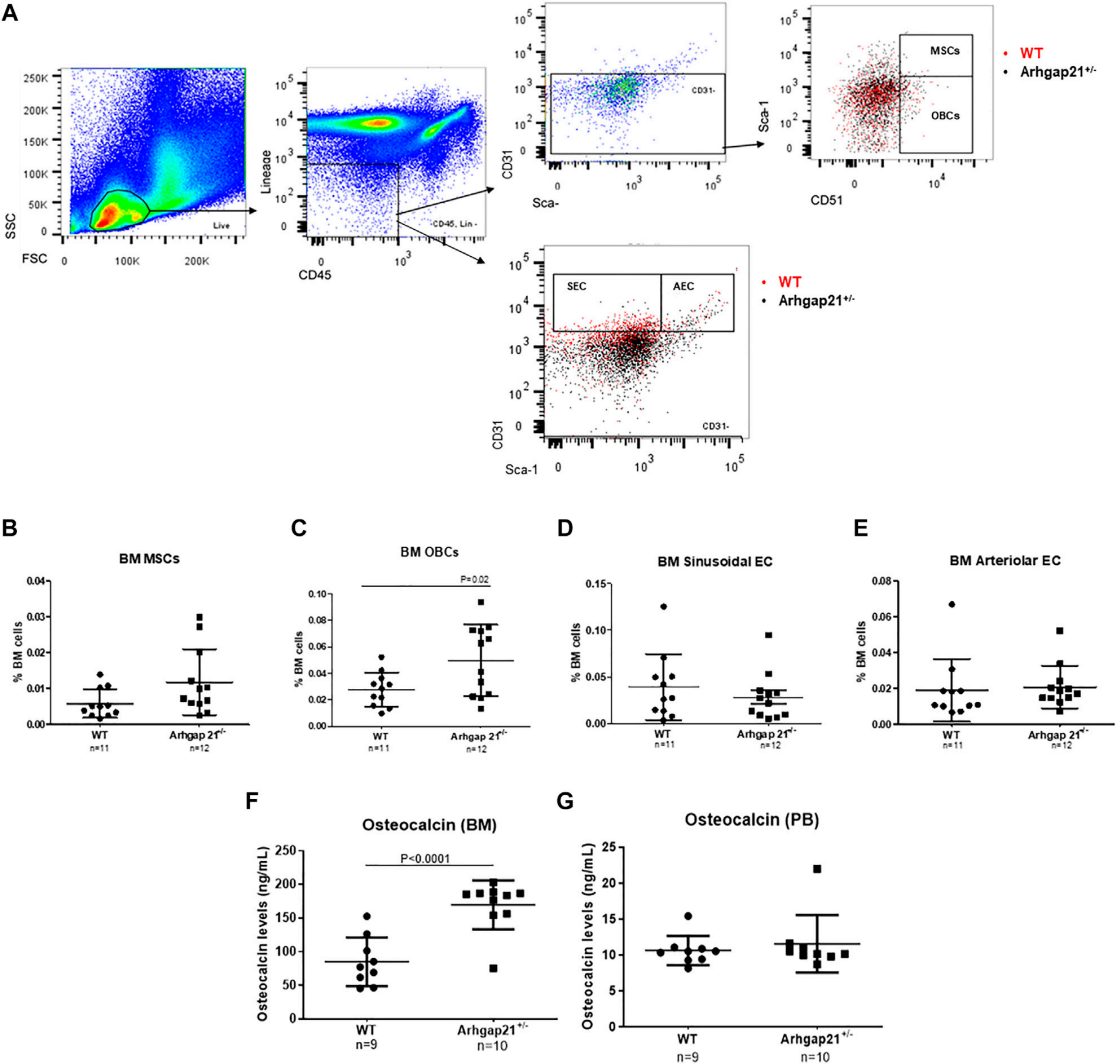
Increased osteoblastic cell population in Arhgap21^+/−^ mice. **(A)** Flow cytometry gating strategy used to identify mesenchymal stromal cells (MSCs), osteoblasts (OBCs), sinusoidal endothelial cells (SEC) and arteriolar endothelial cells (AEC) in murine bone marrow (BM). Last plots show the overlay of wild type (WT in red) and Arhgap21^+/−^ (in black) cellular populations. **(B)** Frequency of MSCs among total bone marrow cells did not significantly differ between WT and Arhgap21^+/−^ mice. **(C)** Arhgap21^+/−^ mice presented increased percentage of BM OBCs compared with WT mice (*p* = 0.02). **(D)** Similar frequencies of BM SEC and **(E)** AEC populations were detected in WT and Arhgap21^+/−^ mice. **(F)** Osteocalcin levels were increased in bone marrow supernatant, **(G)** but not in peripheral blood (PB), from Arhgap21^+/−^ mice compared with WT (*p* < 0.0001). In all graphs, each dot represents an individual mouse; mean and standard error of the mean are shown; 2-way Student’s *t*-test.

Homozygous Arhgap21 knockout mice (Arhgap21^−/−^) were not detected 8 days post-conception. Therefore, we aimed to characterize the BM microenvironment of heterozygous Arhgap21^+/−^ mice, which present an average of approximately 50% reduction of Arhgap21 expression in the BM (Xavier-Ferrucio et al., 2018). We first evaluated the frequency of BM multipotent stromal cell population, osteoblasts, and endothelial cells by immunophenotyping with flow cytometry analysis (Figure 1A). BM cells from Arhgap21^+/−^ mice (*n* = 12) showed no difference in the frequency of MSCs compared to WT (*n* = 11) (Figure 1B). However, Arhgap21^+/−^ BM presented increased phenotypic OBCs (Figure 1C). Similar frequencies of AEC and SEC were found in both mice groups (Figures 1D,E).

Serum level of osteocalcin, which is exclusively synthesized by osteoblasts, is used as a marker of bone formation (Zoch et al., 2016). Osteocalcin levels were then analyzed in the BM supernatant and PB plasma of Arhgap21^+/−^ (*n* = 10) and WT (*n* = 9) mice. BM supernatant of Arhgap21^+/−^ mice presented increased levels of osteocalcin compared to WT (*p* < 0.0001) (Figure 1F), but no difference was observed in PB (Figure 1G).

Osteoblasts synthesize and secrete other signaling molecules in addition to osteocalcin, such as growth factors, cytokines and chemokines to support bone architecture and remodeling (Han et al., 2018). We evaluated the levels of G-CSF, M-CSF, IFN-γ, IL-1α, IL-1β, IL-4, IL-10, IL-17, VEGF-A and TGF-β1 in the BM supernatant of the same mice, but no differences were observed between Arhgap21^+/−^ and WT (Supplementary Figure 1). Among these cytokines, only G-CSF, IL-1α, IL-17 and TGF-β1 were detected in the murine PB with high variation and no statistically significant difference between Arhgap21^+/−^ (*n* = 3) and WT (*n* = 3) (data not shown).

### Expression of Osteogenic Markers is Increased in Arhgap21^+/−^ BM Stromal Cells Submitted to Osteogenic Differentiation *In Vitro*

**FIGURE 2:**
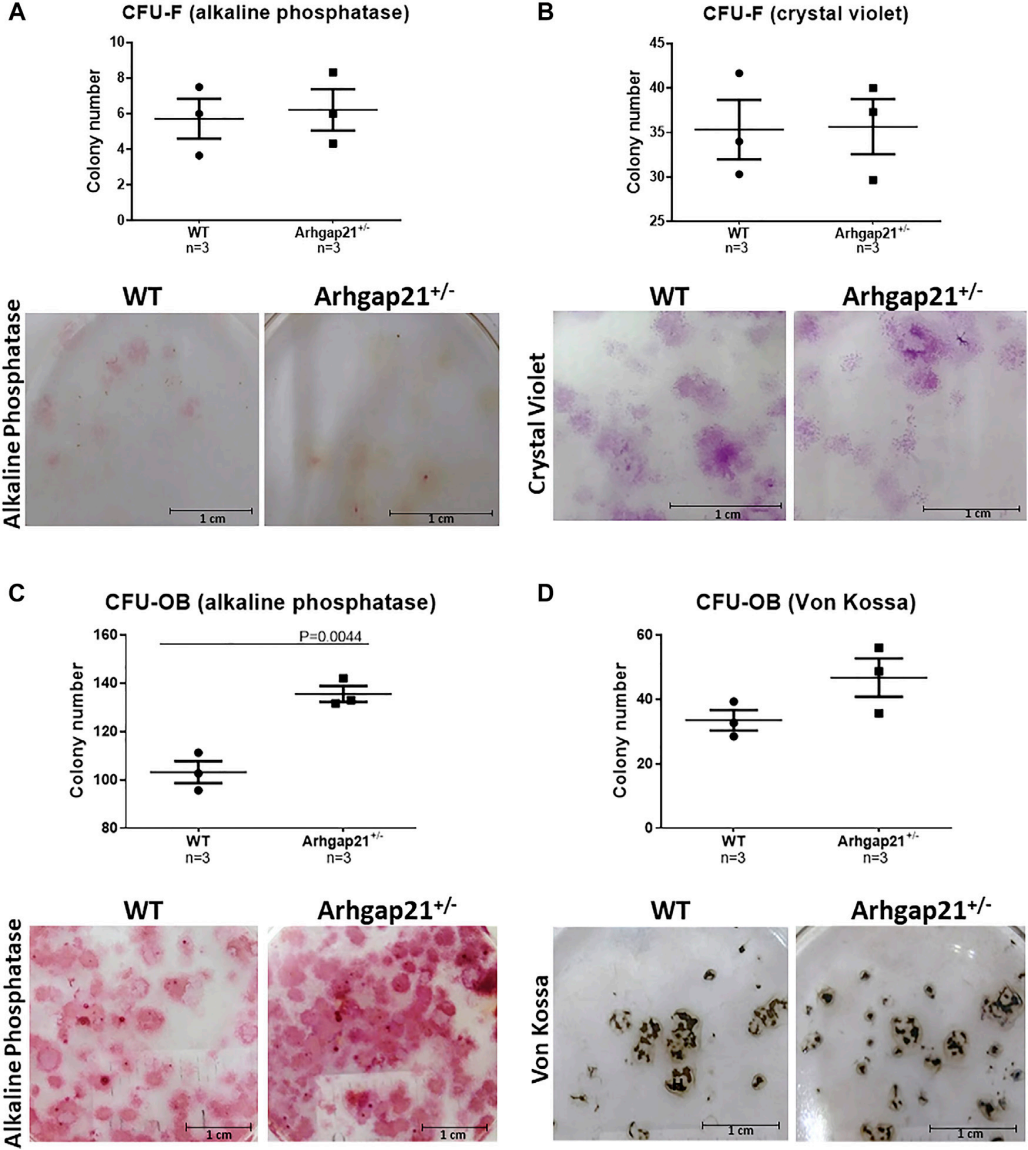
Arhgap21^+/−^ BM cells have a higher potential to form CFU-OB with activity for alkaline phosphatase. **(A)** Number of total CFU fibroblasts (CFU-F) positive stained for alkaline phosphatase or **(B)** crystal violet obtained from WT (*n* = 3) and Arhgap21^+/−^ (*n* = 3) BM cells cultured for a period of 10–14 days. Representative photographs of the stained colonies are shown in the lower panels. **(C)** Number of alkaline phosphatase–positive CFU-OB and **(D)** Von Kossa–positive mineralized nodules formed from BM cells after 17 days of culture under osteoblast conditions. Representative photographs of the stained colonies are shown in the lower panels. We detected a significantly higher number of colonies with alkaline phosphatase activity formed from Arhgap21^+/−^ BM cells compared with WT (*p* = 0.0044). Three independent biological experiments were performed in triplicate for each assay. (In all graphs, each dot represents an individual mouse; mean and standard error of the mean are shown; 2-way Student’s *t*-test.

We next investigated whether the increased frequency of OBC in the Arhgap21^+/−^ BM microenvironment could be caused by enhanced osteogenic differentiation of BM cells. Therefore, we evaluated their ability to form colonies of fibroblasts (CFU-F) and osteoblasts (CFU-OB) *in vitro*. No differences were observed in the numbers of CFU-F (stained positive for alkaline phosphatase or crystal violet) obtained from BM cells of Arhgap21^+/−^ and WT mice (Figures 2A,B). According to the immunophenotyping findings, the number of Arhgap21^+/−^ CFU-OB stained for alkaline phosphatase activity was significantly increased compared to controls (*p* < 0.05) (Figure 2C). An elevated number of von Kossa-positive CFU-OB was also obtained from Arhgap21^+/−^ mice compared to WT, although not statistically significant (Figure 2D).

**FIGURE 3:**
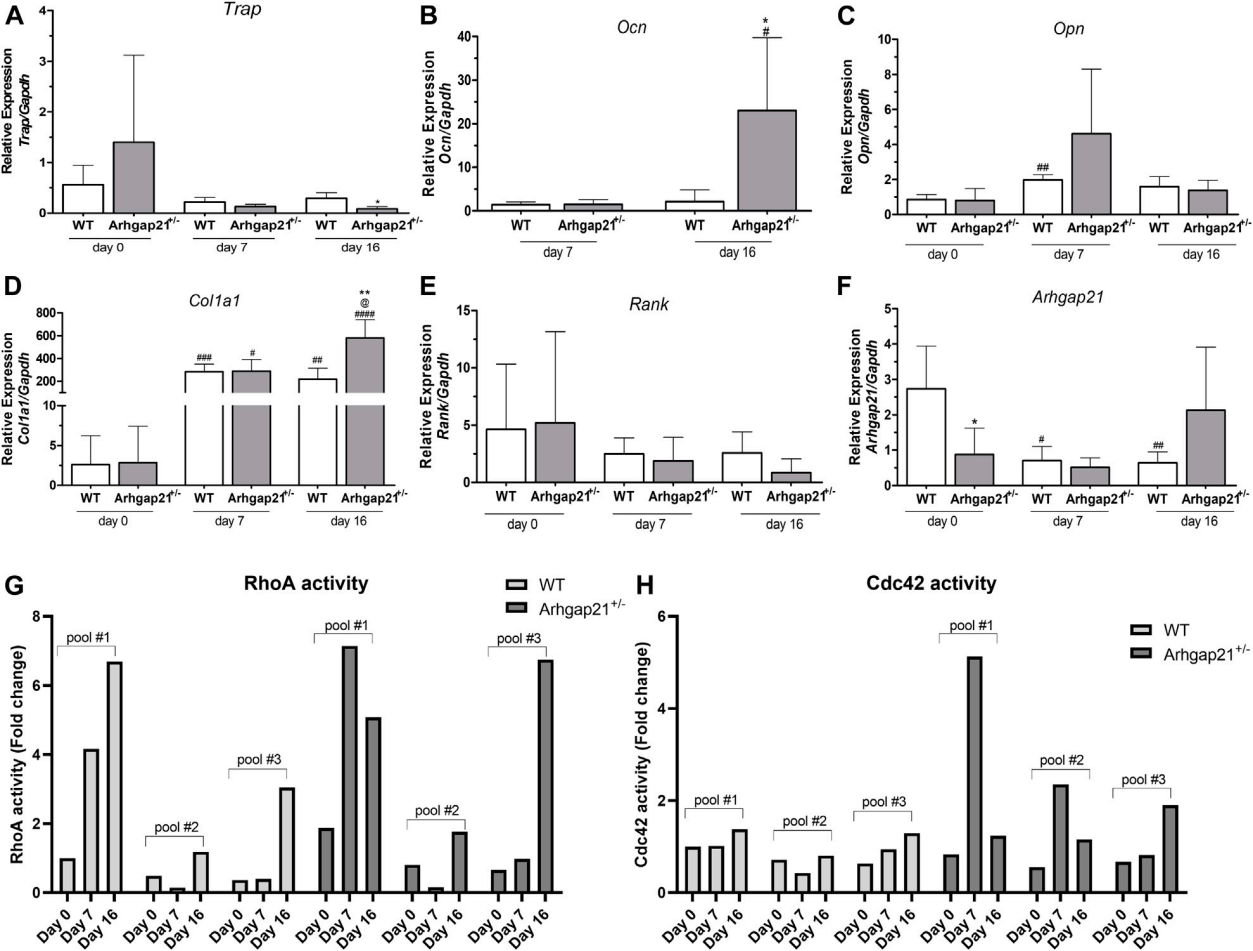
Arhgap21^+/−^ BM cells present increased expression of osteogenic markers when induced for osteoblast differentiation. **(A–F)** Analysis of the mRNA expression of *Col1a1*, *Ocn*, *Opn*, *Trap*, *Rank* and *Arhgap21* before (day 0) and on days 7 and 16 after induction of differentiation (mean ± S.D., *n* = 4). **(A)** Expression of *Col1a1* and **(B)** *Ocn* (both positive regulators of osteoblastic differentiation) was increased in Arhgap21^+/−^ cells compared to WT cells on day 16 of differentiation. *Ocn* expression was not detected on day 0 in both groups. **(D)** Athgap21^+/−^ cultures also showed decreased levels of *Trap* on day 16. Expression of **(C)** *Opn* and **(E)** *Rank* did not significantly differ between Arhgap21^+/−^ and WT during the differentiation, **(F)** whereas Arhgap21 expression was reduced only during differentiation of WT cells. 2-way Student’s *t*-test. **(G)** RhoA and **(H)** Cdc42 activities were also evaluated during osteogenic differentiation of Ahgap21^+/−^ and WT BM cells. RhoA activity seems to increase through the induction of differentiation of both Ahgap21^+/−^ and WT BM cells, whereas Cdc42 activity appears to increase only in Arhgap21^+/−^ cells. RhoA and Cdc42 activities were analyzed in three pools of Arhgap21^+/−^ or WT BM cells containing three animals each.

Expression of osteogenic-associated genes was evaluated before (day 0), and during early (day 7) and late (day 16) stages of osteogenic differentiation. Culturing in osteogenic media induced the expression of collagen type 1 alpha 1 (*Col1a1*), osteocalcin (*Ocn*) and osteoprotegerin (*Opn*). *Ocn*, a marker of late osteogenic differentiation (Amin et al., 2012, 2013), was not detected on day 0, whereas *Opn*, a marker of early osteogenic differentiation (Amin et al., 2012, 2013), was upregulated only on day 7 (Figures 3A–C). Expression of the negative regulators of osteogenic differentiation, tyrosine-rich amelogenin peptide (*Trap*) and nuclear factor κ B (*Rank*) were also analyzed (Figures 3D,E).

In accordance with CFU-OB counts, Arhgap21^+/−^ cultures showed increased mRNA levels of *Col1a1* (mean 580.51 ± SD 159.56 in Arhgap21^+/−^ versus 218.75 ± 96.63 in WT, *p* < 0.01) and *Ocn* (23.06 ± 16.62 in Arhgap21^+/−^ versus 2.16 ± 2.68 in WT, *p* < 0.05) on day 16 (Figures 3A,B). Expression of *Trap1* was decreased in Arhgap21^+/−^ cultures (0.08 ± 0.04 in Arhgap21^+/−^ versus 0.29 ± 0.10 in WT, *p* < 0.05) on day 16 (Figure 3D). No significant differences were observed in the expression of *Opn* (Figure 3C) and *Rank* (Figure 3E) between Arhgap21^+/−^ and WT cultures.

ARHGAP21 was previously described as upregulated during erythroid (Sanchez Bassères et al., 2002) and megakaryocyte (Bernusso et al., 2021) differentiation of human hematopoietic cells. Therefore, we also evaluated whether *Arhgap21* mRNA levels would be altered during osteogenic differentiation. As expected, *Arhgap21* expression was reduced in Arhgap21^+/−^ BM cells compared to WT cells on day 0 of differentiation. Culturing in osteogenic media induced a decrease in *Arhgap21* expression in WT cells (1.73 ± 1.21 on day 0 versus 0.70 ± 0.40 on day 7 and 0.64 ± 0.3 on day 16, *p* < 0.01) but not in Arhgap21^+/−^ cells (Figure 3F).

RhoA and Cdc42 signaling pathways act as positive regulators of MSC osteogenesis (Mcbeath et al., 2004; Gao et al., 2011; Wang et al., 2012; Wang et al., 2012; John et al., 2018). We then investigated the activity of these Rho GTPases during osteogenic differentiation. RhoA activity was increased during differentiation, especially in late differentiation (day 16) in both Arhgap21^+/−^ and WT cultures. Interestingly, Cdc42 activity seemed to increase only during differentiation of Arhgap21^+/−^ cells, whereas no changes were observed during differentiation of WT BM cells.

### Arhgap21^+/−^ Recipient Mouse Showed Reduced Numbers of WBC After BM Transplant

**FIGURE 4:**
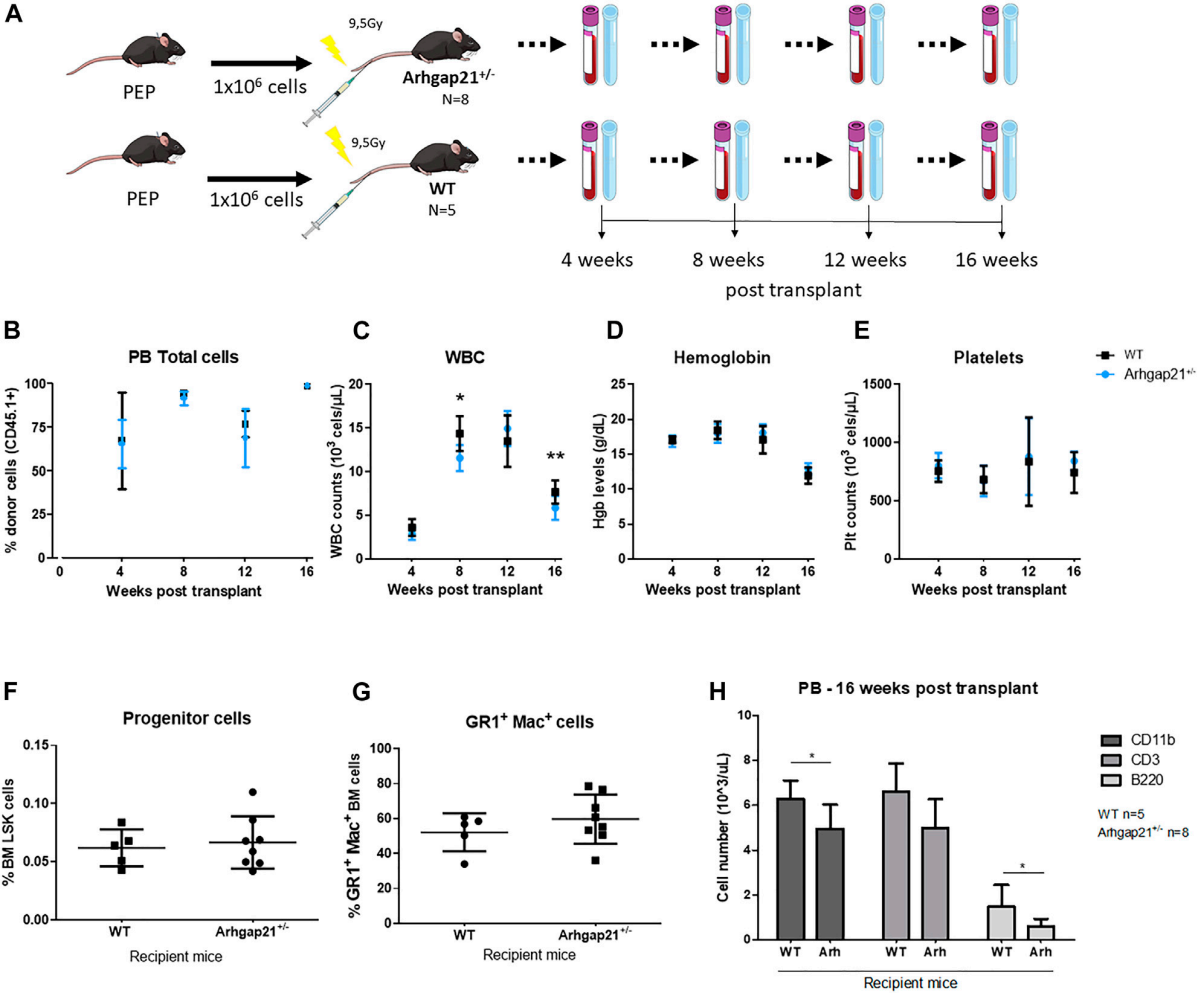
Decreased WBC reconstitution in Arhgap21^+/−^ recipients of bone marrow transplantation. **(A)** Schematic figure of 1 × 10^6^ PEP BM cells transplanted into irradiated (9.5 Gy) Arhgap21^+/−^ recipients (*N* = 8) and WT recipients (*N* = 5). Recipient mice were maintained for 16 weeks after transplantation and chimerism (CD45.1+), WBC count, hemoglobin levels (Hgb) and platelet count (Plt) were assessed every 4 weeks post-transplant. **(B)** Engraftment of PEP marrow cells CD45.1+ were similar in WT and Arhgap21^+/−^ recipients. **(C)** WBC counts were decreased in Arhgap21^+/−^ recipients compared with WT recipients at weeks 8 and 16 following the transplant. **(D)** Hemoglobin levels and **(E)** platelet counts did not differ between WT and Arhgap21^+/−^ recipients. **(F)** Frequency of immature LSK and **(G)** GR1^+^ Mac^+^ cellular populations were also similar between the two groups. **(H)** Absolute counts of CD11b+ and B220 + cells (but not CD3^+^ cells) were decreased in the PB of Arhgap21^+/−^ recipients compared with WT recipients. Mean and standard error of the mean are shown in all graphs; 2-way Student’s *t*-test; *p < 0.05.

Osteoblasts have been shown to regulate the number and localization of transplanted HSC in the BM (Calvi et al., 2003; Zhang et al., 2003; Ho and Méndez-Ferrer 2020). Therefore, we investigated whether the increased population of OBCs in the Arhgap21^+/−^ BM niche would interfere in hematopoietic reconstitution. Accordingly, we performed BM transplants with wild-type CD45.1 hematopoietic stem and progenitor cells (HSPC) into conditioned Arhgap21^+/−^ or wild-type recipient mice (both CD45.2) (Figure 4A). Transplant efficiency was tracked every 4 weeks post-transplantation until 16 weeks, when mice were terminated. No difference was observed in the chimerism of Arhgap21^+/−^ and WT recipients (Figure 4B). Arhgap21^+/−^ recipient mice showed significantly reduced levels of WBC compared to WT after 8 (*p* < 0.01) and 16 (*p* < 0.05) weeks post-transplant (Figure 4C). Hemoglobin levels and platelet counts did not differ between Arhgap21^+/−^ and WT recipient mice either (Figures 4D,E). Sixteen weeks post-transplant, the BM from Arhgap21^+/−^ and WT recipients was analyzed by flow cytometry and no statistically significant differences were observed in the frequencies of LSK (Figure 4F), GR-1+Mac-1+ cells (Figure 4G). Absolute counts of CD11b+ and B220+ cells were decreased in the PB of Arhgap21^+/−^ recipients compared to WT group (*p* < 0.05), whereas no differences were detected in the numbers of CD3^+^ cells (Figure 4H).

### *ARHGAP21* Gene Expression Is Increased in Mesenchymal Stromal Cells From AML Patients

Besides playing an important role in regulating normal HSCs, the osteogenic niche has been shown to participate in the pathogenesis of MDS and AML. Genetic depletion of osteoblasts in AML mouse models increased tumor engraftment and blast numbers leading to shorter survival. Conversely, maintenance of the osteoblast pool resulted in reduced tumor burden and longer survival (Krevvata et al., 2014). AML patients present inhibition of osteogenesis associated with impaired expression of genes related to bone growth and mineralization in the BM, concomitant with decreased levels of osteocalcin in PB (Chen et al., 2020). Therefore, we wondered whether *ARHGAP21* gene expression would be altered in MSCs from MDS and AML patients.

MSCs from *de novo* AML patients [median 1.50 (range 0.43–3.69)] presented higher ARHGAP21 expression compared to MSCs from HD [0.13 (0.07–1)], *p* = 0.0035. ARHGAP21 expression was also increased in MSCs from *de novo* AML when compared to MDS [0.20 (0.00–1.65)], *p* = 0.0001, and AML-MRC patients [0.22 (0.43–0.32)], *p* = 0.0009. No statistical differences in ARHGAP21 expression were observed between the two groups of MDS patients classified according to the WHO-2016 [RS-SLD/RS-MLD/del(5q)/SLD/MLD versus EB-1/EB-2] (Supplementary Figure 2A). Correspondingly, ARHGAP21 expression was increased in total bone marrow samples from de novo AML patients compared to MDS patients classified according to WHO classification [RS-SLD/RS-MLD/del(5q)/SLD/MLD (*p* = 0.0036) and EB-1/EB-2 (*p* = 0.0093)] (Supplementary Figure 2B).

## Discussion

Rho GTPases control several aspects of hematopoiesis, including the interactions between HSPC and other components of the BM microenvironment (Jansen et al., 2005; Yang et al., 2007; Cancelas and Williams 2009; Jaganathan et al., 2013). However, little is known about the participation of Rho GTPase regulators in the hematopoietic niche. Results from this study indicate that the RhoGAP Arhgap21 is important for a proper composition of the BM microenvironment.

The bone marrow of Arhgap21 heterozygous knockout mouse (Arhgap21^+/−^) presented expansion of osteoblastic lineage cells and increased levels of osteocalcin, pointing out a role of Arhgap21 in osteogenic niche. Notably, these changes are not homogeneous within the Arhgap21^+/−^ mice and phenotypic variation was already reported for other hematological parameters in Arhgap21^+/−^ individuals (Xavier-Ferrucio et al., 2018). High phenotypic heterogeneity is frequently observed in other transgenic mice, including heterozygous knockout mouse models for other RhoGAPs and mouse models for hematological malignancies (Raman et al., 2013; Balderman et al., 2016; McMichael et al., 2017; Lovat et al., 2018; Almosailleakh and Schwaller 2019). Osteoblast ablation in mice decreased the overall BM cellularity and altered the number of long-term HSC (LT-HSC) subtypes. HSC quiescence and long-term engraftment were also reduced by osteoblast depletion (Bowers et al., 2015). Our previous work revealed that Arhgap21^+/−^ mice present increased frequency of phenotypic HSC with decreased ability to form colonies *in vitro* and *in vivo* and to engraft during serial transplantation (Xavier-Ferrucio et al., 2018). These alterations in Arhgap21^+/−^ HSCs possibly result from a combination of intrinsic and extrinsic changes triggered by Arhgap21 reduction, such as modifications in the local microenvironment. Development of mouse models with cell type conditional Arhgap21 deletion will be important in assessing to what extent the HSPC alterations are due to the unbalanced microenvironment or the inverse. Notably, we herein observed a decreased WBC reconstitution when normal hematopoietic cells were transplanted into Arhgap21^+/−^ recipients, possibly due to a decrease in monocytes/granulocytes and B lymphocytes. No differences were detected in the percentage of BM progenitor cells (LSK cells) neither in BM GR1+Mac + cells between Arhgap21^+/−^ and WT recipients, suggesting that homing to the Arhgap21^+/−^ BM niche was not altered and that the reduction in PB WBC is related to the differentiation process.

The higher frequency of osteoblasts may be caused by increased capacity of Arhgap21^+/−^ BM cells to form functional osteoprogenitors (CFU-OB positive for alkaline phosphatase). Arhgap21^+/−^ BM cells also presented enhanced expression of *Col1a1* and *Ocn* when submitted to osteogenic differentiation. Type I collagen is the most abundant protein of the bone extracellular matrix and expression of *Col1a1* is detected at early periods of osteogenic differentiation (Dacic et al., 2001). Ocn is exclusively produced by osteoblast lineage cells and is detected at late stages of differentiation (Dacic et al., 2001). Increased mRNA levels of both *Col1a1* and *Ocn* were detected on day 16 of differentiation, indicating that Arhgap21 deficiency affects the late stages of differentiation. Arhgap21^+/−^ cells also showed decreased mRNA levels of *Trap* (*Acp5*), a marker of osteoclast activity, in the end of differentiation possibly as a result the of fate decision towards osteoblast lineage.

The activity of Cdc42 seems to increase during osteogenic differentiation of Arhgap21^+/−^, but not in WT cells, which is in accordance with previous studies that showed increased Cdc42 upon ARHGAP21 inhibition (Dubois et al., 2005; Bigarella et al., 2009). Activation of Cdc42 signaling has been shown to enhance MSCs osteogenesis through the control of cell shape and cytoskeletal rearrangements required for differentiation (Gao et al., 2011; John et al., 2018). Therefore, the increased activity of Cdc42 in Arhgap21^+/−^ cells may be a possible mechanism triggering higher osteoblast differentiation. RhoA activity was increased during osteogenic differentiation of Arhgap21^+/−^ and WT cells, which is in agreement with previous studies (Arnsdorf et al., 2009; Zhang et al., 2019). However, no differences in RhoA activity were found between Arhgap21^+/−^ and WT cells during differentiation.

There is increasing evidence that alterations in the BM microenvironment may facilitate the development of hematological neoplasia, which can originate in the niche itself (Kode et al., 2014; Méndez-Ferrer et al., 2020). We have previously demonstrated that MSCs derived from *de novo* AML patients present reduced growth rate and impaired immunosuppressive ability with a distinct cytokine expression profile (Lopes et al., 2017). Moreover, the osteoblast niche supposedly hides quiescent leukemia stem cells, cooperating for chemoresistance and relapse (Méndez-Ferrer et al., 2020). Interesting, we found increased ARHGAP21 gene expression in MSCs derived from BM cells of *de novo* AML patients in comparison with HD and patients with MDS or AML developed after myelodysplasia. Recent findings showed that AML MSCs present decreased expression of osteogenesis-related genes and impaired osteogenic differentiation capacity (Geyh et al., 2016; Xu et al., 2020). Lower levels of osteocalcin were also found in the PB of AML patients compared to non-leukemic subjects and predicted poorer clinical outcome (Chen et al., 2020). The elucidation of the effects of ARHGAP21 overexpression in AML niche is still pending, but we speculate that it is related to decreased osteogenesis reported in these patients.

In conclusion, our study revealed a novel function of Arhgap21 in the BM microenvironment. Using a heterozygous Arhgap21 knockout mouse model, we showed that decreased expression of this RhoGAP led to the expansion of BM osteoblastic niche, which may be associated with several hematological defects previously reported. ARHGAP21 also may participate in the deregulation of the leukemic microenvironment and could be investigated as a possible therapeutic target.

## Data Availability

The original contributions presented in the study are included in the article/Supplementary Material, further inquiries can be directed to the corresponding authors.
