# Supplementary material for: Arhgap21 Deficiency Results in Increase of Osteoblastic Lineage Cells in the Murine Bone Marrow Microenvironment
Source: Front Cell Dev Biol. 2021 Nov 30;9:718560. doi: 10.3389/fcell.2021.718560 (PMC8670086; doi:10.3389/fcell.2021.718560)
Supplement: Supplementary file 1 [file DataSheet1.pdf]

Supplementary Material

Supplementary Table 1. Patient characteristics

	BM total cells	MSCs
**Patients**	**Number**	**Number**
**MDS**	**48**	**21**
Gender<br>Male/Female	27/23	15/6
Age (years), media (range)	70 (15-86)	70 (16-90)
WHO 2016 classification		
RS-SLD / RS-MLD / del(5q) / SLD / MLD	1/11/1/0/20	3/1/0/1/10
EB-1/EB-2	5/10	2/4
R-IPSS		
Very low / Low	6 / 20	2/7
Intermediate	9	7
High / Very high	7 / 3	1/3
Not available	3	1
Cytogenetic risk[1]		
Good	36	15
Intermediate	8	2
Poor	1	3
No growth	3	1
**AML**	**50**	**18**
de novo AML / AML-MRC	42/8	12/6
Gender<br>Male/Female	33/17	9/9
Age (years), median (range)	69 (22-90)	66 (30-86)
BM blasts (%), median (range)	70 (22-96)	50 (20-89)
Cytogenetic risk[2]		
Good	5	10
Intermediate/Poor	26/9	0/8
No growth	10	0

Abbreviations: MDS, myelodysplastic syndromes; MDS-MLD: MDS with multilineage dysplasia; MDS-SLD: MDS with single lineage dysplasia; MDS-RS: MDS with ring sideroblasts; MDS-EB: MDS with excess blasts; MDS with isolated del(5q); WHO, World Health Organization; R-IPSS, Revised International Prognostic Scoring System; BM, bone marrow; AML, acute myeloid leukemia; AML-MRC, acute myeloid leukemia with myelodysplasia-related changes. [1]Cytogenetic risk for MDS was defined according to R-IPSS (Arber et al. 2016). [2]Cytogenetic risk for AML was defined according to Grimwade et al. (Grimwade et al. 2016)

Supplementary Table 2. Sequence and concentration of primers used for quantitative PCR

Gene	Primers (5' – 3')	Product length	Concentration
ARHGAP21 (human)	AGGCAAACTTTGCTTGGTGCTA<br>ACTGAGAAGTTTCCTTTCCGACTC	87	300nM
HPRT (human)	GAACGTCTTGCTCGAGATGTGA<br>TCCAGCAGGTCAGCAAAGAAT	101	150nM
Arhgap21 (mouse)	GAGGAAAGCTTCAAGCACCA<br>GATGACAGCAGATGCAGGAA	121<br>121	150nM
Col1a1 (mouse)	AGCACGTCTGGTTTGGAGAG<br>GACATTAGGCGCAGGAAGGT	112	150nM
Ocn (mouse)	GGCCCAGACCTAGCAGACAC<br>CTGGGCTTGGCATCTGTGAG	98	150nM
Opn (mouse)	TGGCTGAATTCTGAGGGACTAAC<br>TATAGGATCTGGGTGCAGGCT	150<br>147	150nM
Rank (mouse)	TTGCACGGCTGGCTACCACT<br>GCACACCGTATCCTTGTTGAGCTG	115	150nM
Trap (mouse)	AGCCACATACGGGGTCACTG<br>TAGCCCACACCGTTCTCGTC	81	150nM
Gapdh (mouse)	TGACCACCAACAACTGCTTA<br>GGATGCAGGGATGATGTTC	179	150nM

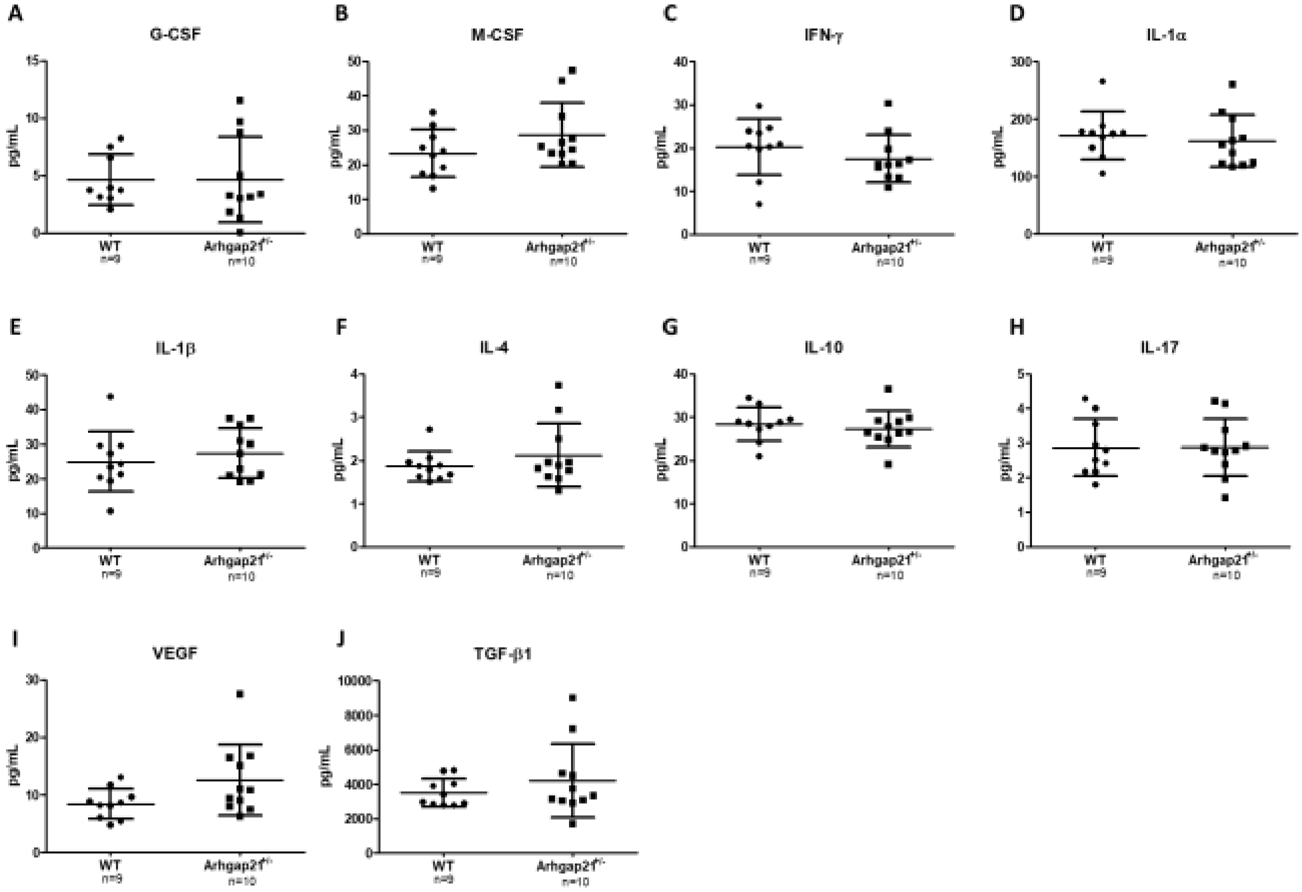

Supplementary Figure 1. Levels of IFN-γ, M-CSF, G-CSF, TGF-β1, IL-1α, IL-1β, IL-4, IL-10, IL-17, and VEGF-A in the bone marrow supernatant measured using Luminex xMAP assay. No difference was found in the levels of the tested cytokines between bone marrow supernatant from Arhgap21+/- and WT. Each dot represents an individual mouse. For all graphs 2-tailed Student's t test statistical analysis were used, mean and standard error of the mean are shown.

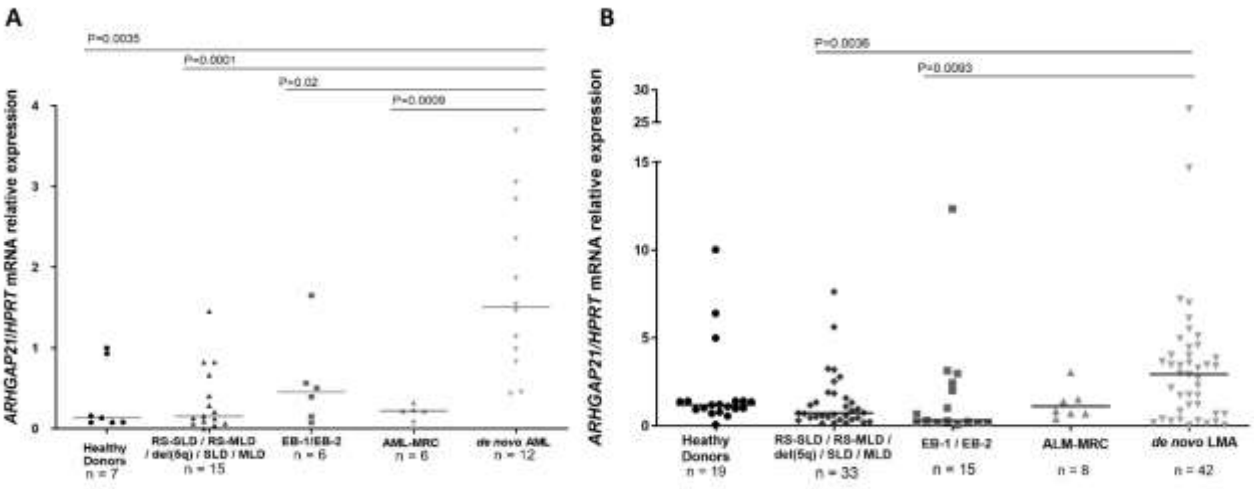

Supplementary Figure 2. Increased expression of ARHGAP21 mRNA in MSCs and total bone marrow samples from *de novo* AML patients. Quantitative PCR analysis of ARHGAP21 expression in MSCs (A) and (B) total bone marrow samples from healthy donors and patients with MDS (RS-SLD, RS-MLD, del(5q), SLD, MLD), MDS (EB-1, EB-2), AML-MRC and *de novo* AML. Each dot represents one subject and horizontal lines indicate medians. The number of subjects and P values are indicated (Mann–Whitney test). mRNA expression levels of *ARHGAP21* were normalized by *HPRT* endogenous control.

References

Arber DA, Orazi A, Hasserjian R, Borowitz MJ, Beau MM Le, Bloomfield CD, et al. The 2016 revision to the World Health Organization classi fi cation of myeloid neoplasms and acute leukemia. Blood. 2016;127(20):2391–406.

Grimwade D, Ivey A, Huntly BJP. Molecular landscape of acute myeloid leukemia in younger adults and its clinical relevance. Vol. 127, Blood. 2016. p. 29–41.
